# Unexpected identification of obesity-associated mutations in *LEP* and *MC4R* genes in patients with anorexia nervosa

**DOI:** 10.1038/s41598-024-57517-w

**Published:** 2024-03-25

**Authors:** Luisa Sophie Rajcsanyi, Yiran Zheng, Beate Herpertz-Dahlmann, Jochen Seitz, Martina de Zwaan, Wolfgang Herzog, Stefan Ehrlich, Stephan Zipfel, Katrin Giel, Karin Egberts, Roland Burghardt, Manuel Föcker, Jochen Antel, Pamela Fischer-Posovszky, Johannes Hebebrand, Anke Hinney

**Affiliations:** 1 Department of Child and Adolescent Psychiatry, Psychosomatics and Psychotherapy, University Hospital Essen, University of Duisburg-Essen, Virchowstraße 174, 45147 Essen, Germany; 2 Center for Translational Neuro- and Behavioural Sciences, University Hospital Essen, Essen, Germany; 3 Section for Molecular Genetics of Mental Disorders, University Hospital Essen, Essen, Germany; 4 Department of Child and Adolescent Psychiatry and Psychotherapy, University Hospital of the RWTH Aachen, Aachen, Germany; 5 Department of Psychosomatic Medicine and Psychotherapy, Hannover Medical School, Hannover, Germany; 6 Department of Internal Medicine II, General Internal and Psychosomatic Medicine, University of Heidelberg, Heidelberg, Germany; 7 Eating Disorders Research and Treatment Center, Department of Child and Adolescent Psychiatry, Faculty of Medicine, TU Dresden, Dresden, Germany; 8 Translational Developmental Neuroscience Section, Division of Psychological and Social Medicine and Developmental Neurosciences, Faculty of Medicine, TU Dresden, Dresden, Germany; 9 Department of Psychosomatic Medicine and Psychotherapy, Medical University Hospital Tübingen, Tübingen, Germany; 10 Center of Excellence in Eating Disorders KOMET, Tübingen, Germany; 11 Department of Child and Adolescent Psychiatry, Psychosomatics and Psychotherapy, University Hospital Würzburg, Würzburg, Germany; 12 Oberberg Clinic for Child and Adolescent Psychiatry, Psychosomatics and Psychotherapy, Fasanenkiez, Berlin, Germany; 13 Department of Child and Adolescent Psychiatry, University Hospital Münster, Munster, Germany; 14 LWL-University Hospital Hamm for Child and Adolescent Psychiatry, Ruhr-University Bochum, Hamm, Germany; 15 Department of Pediatrics and Adolescent Medicine, University of Ulm, Ulm, Germany; 16 Institute of Sex- and Gender-Sensitive Medicine, University Hospital Essen, Essen, Germany

**Keywords:** Genotype, Medical genetics, Mutation, Sequencing

## Abstract

Mutations leading to a reduced or loss of function in genes of the leptin-melanocortin system confer a risk for monogenic forms of obesity. Yet, gain of function variants in the melanocortin-4-receptor (*MC4R*) gene predispose to a lower BMI. In individuals with reduced body weight, we thus expected mutations leading to an enhanced function in the respective genes, like leptin (*LEP)* and *MC4R*. Therefore, we have Sanger sequenced the coding regions of *LEP* and *MC4R* in 462 female patients with anorexia nervosa (AN), and 445 healthy-lean controls. In total, we have observed four and eight variants in *LEP* and *MC4R*, respectively. Previous studies showed different functional in vitro effects for the detected frameshift and non-synonymous variants: (1) *LEP*: reduced/loss of function (p.Val94Met), (2) *MC4R*: gain of function (p.Val103Ile, p.Ile251Leu), reduced or loss of function (p.Thr112Met, p.Ser127Leu, p.Leu211*fs*X) and without functional in vitro data (p.Val50Leut). In *LEP*, the variant p.Val94Met was detected in one patient with AN. For *MC4R* variants, one patient with AN carried the frameshift variant p.Leu211*fs*X. One patient with AN was heterozygous for two variants at the *MC4R* (p.Val103Ile and p.Ser127Leu). All other functionally relevant variants were detected in similar frequencies in patients with AN and lean individuals.

## Introduction

Anorexia nervosa (AN) is marked by a diminished body weight, a pronounced fear of gaining weight and a distorted body image^1^. Among psychiatric disorders, AN exhibits the highest mortality rates^2–7^. Formal genetic studies revealed a robust genetic component for AN. In fact, twin-based studies have estimated a heritability for AN between 28 and 74%, depending on stringency of its definition and sample size^8^. In the last decade, genome-wide association studies (GWAS) uncovered eight genetic loci associated with AN^9^.

Energy homeostasis and consequently, body weight regulation, are modulated by the leptin-melanocortin-system exerting its functions in the hypothalamus. Leptin (LEP) is secreted by the adipose tissue (AT) and relays adaptive signals about the mass of the AT and the nutritional status to the brain. In the hypothalamic arcuate nucleus, leptin binds to the leptin receptor leading to the inhibition of the orexigenic agouti-related peptide (AgRP)/neuropeptide Y (NPY) neurons, while stimulating the anorexigenic pro-opiomelanocortin (POMC)/cocaine and amphetamine regulated transcript (CART) neurons. The released POMC is post-transcriptionally processed into α-melanocyte-stimulating hormone (α-MSH), which subsequently bind to the melanocortin-4-receptor (MC4R). The resultant activation of MC4R leads to the production of satiety signals and consequently to a lower food intake and higher energy expenditure^10–13^.

Disruption of this regulation by loss of function (LoF) variants is known to be associated with severe monogenic forms of obesity^13,14^. The variants include non-synonymous and nonsense *LEP* variants^15–20^. Yet, gain of function (GoF) variants, e.g. in *MC4R*^21–23^, predispose to a lower body mass index (BMI). Further, previous studies have reported shared genetic loci for reduced BMI and AN risk^24–26^.

Generally, patients with acute AN have considerably reduced serum leptin levels compared to BMI-matched controls. An increase in weight is accompanied by an increase in leptin^27–30^. Besides, a Mendelian randomization study concluded that low leptin levels are correlated with a higher risk of AN^31^. Treating patients with AN off-label with recombinant leptin improved cognition, behaviour and mood^32–34^.

Despite emerging evidence for the crucial role of the leptin-melanocortin system in the aetiology of AN^31,35^, no associations of variants in *LEP* in patients with AN have been described so far^9,36,37^. In *MC4R*, the mutant allele of the variant rs2229616 was previously shown to have a BMI-decreasing effect^21,38^. Previously, we have screened both genes in rather small study groups of patients with AN^36,39^. In the present study, we expanded our previous findings by Sanger sequencing of the coding regions of *LEP* and *MC4R* in larger study groups comprising 462 acutely ill or recovered women with AN and 445 healthy-lean controls. Based on in silico analyses and previously published in vitro and in vivo data, we subsequently assessed the functional consequences of the detected variants.

## Results

### Variants in the coding regions of *LEP*

The sequencing of the coding region of *LEP* revealed a total of four rare (minor allele frequency (MAF) ≤ 0.01%) variants (see Table 1). All identified carriers are heterozygous for the respective variant.

**Table 1:** Minor allele frequencies of the detected variants in *LEP* and *MC4R*.

Gene	Variant	Reference allele	Minor allele	Amino acid exchange	Patients with AN				Healthy-lean individuals				European females in gnomAD
					11	12	22	MAF	11	12	22	MAF	MAF
*LEP*	rs201523305	C	T	Cys7=	461	1	0	0.001	445	0	0	0	0.0002
	rs13306517	A	G	Gln25=	462	0	0	0	444	1	0	0.001	0.0009
	No rsID g.128254532	C	T	Ser91=	461	1	0	0.001	445	0	0	0	*NA*
	rs17151919	G	A	Val94Met	461	1	0	0.001	445	0	0	0	0.0005
*MC4R*	rs121913557	C	A	Val50Leu	461	1	0	0.001	444	1	0	0.001	0
	rs2229616^a^	C	T	Val103Ile	447	15	0	0.02	432	13	0	0.015	0.02
	rs13447329	G	A	Thr112Met	461	1	0	0.001	443	2	0	0.002	0.002
	rs13447331^a^	G	A	Ser127Leu	461	1	0	0.001	445	0	0	0	0.0002
	Novel variant g.60371891	A	G	Tyr153=	461	1	0	0.001	445	0	0	0	*NA*
	No rsID g.60371771	G	A	Val193=	462	0	0	0	444	1	0	0.001	*NA*
	rs13447338^b^	GAGA	-	Leu211*fs*X	461	1	0	0.001	445	0	0	0	0.00002
	rs52820871	T	G	Ile251Leu	456	6	0	0.006	437	8	0	0.009	0.01

Here, all detected variants in the coding regions of *LEP* and *MC4R* are represented for the respective study groups. The alleles are assigned based on the forward strand. Additionally, the minor allele frequency of the non-Finnish European females in the Genome Aggregation Database (gnomAD^90^) for each SNP is given. Some genetic variants are not represented in the used version (v2.1.1) of gnomAD (*NA*).

11, homozygous wildtype; 12, heterozygous; 22, homozygous minor allele.

gnomAD, Genome Aggregation Database; MAF, minor allele frequency; *NA*, not available.

^a^One female patient with AN carried two heterozygous variants (rs2229616, p.Val103Ile and rs13447331, p.Ser127Leu). She had a BMI of 15.67 kg/m^2^ at admission, increased weight during her in-patient treatment and was discharged with a BMI of 17.75 kg/m^2^. After 2.5 years, her BMI was 22.66 kg/m^2^. Her leptin levels were 1.66 µg/l at admission, while these increased during in-patient treatment to 5.59 µg/l at discharge. At the 2.5 year follow-up, her leptin levels were 43.31 µg/l.

^b^The patient with AN carrying this variant heterozygously had a BMI of 13.54 kg/m^2^ at admission which increased to 18.56 kg/m^2^ at discharge. At the follow-up time point of 2.5 years, her BMI was 18.29 kg/m^2^. Her leptin levels were at admission: 0.85 µg/l, at discharge 10.6 µg/l and at the follow-up time point 6.61 µg/l.

One variant led to a non-conservative amino acid exchange (rs17151919, p.Val94Met), while all others were synonymous variants (rs201523305, p.Cys7=; rs13306517, p.Gln25= and a variant without an rsID, p.Ser91=). The non-synonymous variant (rs17151919) was detected in the heterozygous state in an acutely ill female patient with AN (age: 34.12 years old; BMI: 15.94 kg/m^2^; BMI-SDS_LMS_: − 3.62). In silico tools, like MutationTaster2021^40^ and PredictSNP2^41^, hinted at an overall non-pathogenic effect. Yet, the resultant amino acid substitution was predicted to have a destabilizing impact on LEP (see Table 2). Previously, two studies have implied a role of the mutant allele (Met94; in mature protein this equals position 73; see Supplementary Fig. 1) in body weight regulation^42,43^. Met94 was already reported to be associated with lower leptin levels and a higher childhood BMI in individuals of African ancestry^42^. At baseline, each mutant allele added approximately 2.6 kg to the carrier’s weight, while the weight increased to 4.8 kg at year 20^43^. No associations were detected in Europeans, as this variant was extremely rare in this population^42^. Functional in vitro analyses reported a decreased leptin secretion in human embryonic kidney 293 cells caused by the substitution of valine by methionine^42^.

**Table 2:** In silico analyses for the detected variants in *LEP* and *MC4R*.

Gene	Variant	Amino acid exchange	Predictions for all variants			Predictions for non-synonymous variants			
			ESEfinder	Mutation Taster2021	PredictSNP2	PANTHER-PSEP	CUPSAT		SIFT
			Splice site	Prediction	Prediction	Prediction	Stability	Torsion	Prediction
*LEP*	rs201523305	Cys7=	Changed	Benign	Neutral	–	–	–	–
	rs13306517	Gln25=	Not changed	Benign	Neutral	–	–	–	–
	No rsID g.128254532C/T	Ser91=	Not changed	Benign	Neutral	–	–	–	–
	rs17151919	Val94Met	Not changed	Benign	Neutral	Probably benign	Destabilizing	Favourable	Tolerated
*MC4R*	rs121913557	Val50Leu	Changed	Deleterious	Deleterious	Probably benign	Destabilizing	Favourable	Deleterious
	rs2229616	Val103Ile	Changed	Benign	Deleterious	Probably benign	Destabilizing	Favourable	Tolerated
	rs13447329	Thr112Met	Changed	Benign	Neutral	Probably benign	–	–	Tolerated
	rs13447331	Ser127Leu	Changed	Deleterious	Deleterious	Probably damaging	Stabilizing	Favourable	Tolerated
	Novel variant g.60371891A/G	Tyr153=	Changed	Benign	Neutral	–	–	–	–
	No rsID g.60371771G/A	Val193=	Not changed	Benign	Neutral	–	–	–	–
	rs13447338	Leu211fsX	Changed	–	–	–	–	–	–
	rs52820871	Ile251Leu	Changed	Benign	Neutral	Probably benign	Destabilizing	Favourable	Tolerated

To predict putative effects of the mutant alleles, various in silico tools were applied. Putative impacts on splicing products were examined with the tool ESEfinder^83^. The general pathogenicity was predicted with MutationTaster2021^40^ and PredictSNP2^41^. The latter being a consensus classifier condensing results of multiple tools, like CADD and FATHMM. Indications for impacts on the proteins were investigated with PANTHER-PSEP^84^, CUPSAT^85^ and SIFT^86^. Tools predicting effects on the protein were only applicable for non-synonymous variants. Yet, as the used protein reference sequence of MC4R was incomplete, the non-synonymous variant rs13447329 (p.Thr112Met) could not be analysed with CUPSAT and SIFT.

Moreover, three synonymous variants, namely rs201523305 (p.Cys7=), rs13306517 (p.Gln25=) and a variant without an assigned rsID (p.Ser91=), were determined (see Table 1). The latter variant (p.Ser91=) had already been detected in our previous mutation screen^36^. One recovered patient with AN (BMI: 21.23 kg/m^2^; BMI-SDS_LMS_: − 1.11) harboured the variant rs201523305 (p.Cys7=) predicted to alter splice sites (see Table 2). The variant leading to p.Ser91 = was found in one acutely ill adolescent with AN (BMI: 15.43 kg/m^2^; BMI-SDS_LMS_: − 3.03). Again, in silico tools hinted at a benign impact (see Table 2). The variant rs13306517 (p.Gln25=) was exclusively detected in one lean female control (see Table 1). This variant was implied not to have functional implications (see Table 2). Beyond, no functional studies have previously been published for these variants (see Supplementary Tables 2 and 4).

### Variants in the coding region of *MC4R*

Eight variants located in the coding sequence of *MC4R* were identified (see Table 1). Five of those are non-synonymous variants (rs121913557, p.Val50Leu; rs2229616, p.Val103Ile; rs13447329, p.Thr112Met; rs13447331, p.Ser127Leu; rs52820871, p.Ile251Leu; for structural positions of non-synonymous variants see Supplementary Fig. 2). Additionally, one frameshift (rs13447338, p.Leu211*fs*X), one novel synonymous (p.Tyr153=) and one synonymous variant without an assigned rsID (p.Val193=) were determined (see Table 1).

We observed the non-synonymous variants rs2229616 (p.Val103Ile) and rs52820871 (p.Ile251Leu) in similar frequencies in our 462 patients with AN, and 445 healthy-lean controls (see Table 1). Within our control group, which included both sexes, variant rs52820871 (p.Ile251Leu) was detected four times in each sex, while variant rs2229616 (p.Val103Ile) was present in four males and nine females. Our in silico analyses hinted at a putative deleterious effect caused by rs2229616 without affecting splice sites and the protein’s torsion, while rs52828071 was predicted to be overall benign but has the potential to destabilize MC4R (see Table 2). In our literature search, we found numerous studies reporting a reduced risk for obesity and an association with a lower BMI of the infrequent alleles of rs2229616 and rs52820871^21,22,44^. In 2004, Geller et al.^21^ detected a reduced transmission rate of the Ile103 allele in an analysis of family-based samples, thus providing initial evidence for an obesity-protective effect of this variant. Functional implications of both frequent variants have been thoroughly studied, as well. For instance, the introduction of the variants in inbred mice, showed no significant difference in food intake and BMI between homozygous or heterozygous mice for each mutant and their wildtype (WT) littermates. Yet, homozygous female mice (*MC4R*^*V103I/V103I*^ and *MC4R*^*I251L/I251L*^) had a reduced longitudinal length and their white adipose tissue weighed less in relation to their WT littermates. Albeit, when fed a high-fat diet, these mice’s adiposity resembled those of their littermates not carrying a variant^23^. Another study showed that variants p.Val103Ile and p.Ile251Leu can be characterised as GoF variants as they have a significant bias towards the ß-arrestin recruitment rather than cyclic adenosine monophosphate (cAMP)-mediated signalling. Beyond, it was determined that the MC4R carrying the mutant Ile103 allele did not internalize like the WT MC4R and consequently exhibited a stable expression on the cell surface upon agonist stimulation^22^. In accordance, another study supported a deviating signalling behaviour due to p.Val103Ile yielding in GoF characteristics^45^. Conflicting to these findings, others reported that both variants displayed an elevated presence at the plasma membrane. Upon agonist stimulation, solely p.Ile251Leu led to a reduced internalisation, while the MC4R with the mutant Ile103 allele maintained an unaltered internalisation^46^. Besides, multiple studies did not ascertain any functional differences between the WT and mutant MC4R for p.Val103Ile and p.Ile251Leu^21,47–50^.

In our mutation screen, we have further detected one lean control individual (BMI: 17.00 kg/m^2^; BMI-SDS: − 2.64) harbouring two frequent variants leading to p.Val103Ile and p.Ile251Leu. Similarly, one patient with AN carried the common rs2229616 (p.Val103Ile) and the rare variant rs13447331 (p.Ser127Leu). This patient had a restrictive AN. At admission the patient had a weight of 45.3 kg being 169.5 cm tall (BMI: 15.67 kg/m^2^; 4th BMI-percentile). During the 19 weeks in-patient treatment 6 kg were gained resulting in a BMI of 17.75 kg/m^2^ at discharge. Leptin levels were 1.66 µg/l at admission and increased during treatment up to 5.59 µg/l at discharge. After 2.5 years, the patient had a BMI of 22.66 kg/m^2^ (weight: 66 kg), while leptin levels were 43.31 µg/l. In vitro studies identified in the subsequent literature search on double mutant receptors with p.Val103Ile and p.Ser127Leu demonstrated that these can cause a reduced efficacy and potency^51^ and are less abundant on cell surfaces^52^. An increase in the cAMP accumulation in relation to the single mutant MC4R carrying the Leu127 allele was determined^52^. Within our mutation analysis, we did not detect the rare variant leading to the non-conservative amino acid substitution from serine to leucine at position 127 in any additional individuals here (see Table 2). Our in silico analyses detected functional implications for this variant, as it was rated as deleterious by MutationTaster2021 and PredictSNP2. Yet, no destabilizing potential on the MC4R protein was indicated (see Table 2).

The frameshift mutation in *MC4R* (rs13447338) resulting in a truncated protein (p.Leu211*fs*X) was detected once in a female adolescent with AN, and not in the lean individuals (see Table 1). The female patient with AN displayed the restrictive form of AN. At admission, she weighed 35.10 kg while being 161 cm tall (BMI: 13.54 kg/m^2^; 0th BMI-percentile). During her 20-week in-patient treatment, she gained 13 kg. At discharge, she had a weight of 48.1 kg and a BMI of 18.56 kg/m^2^. Leptin levels were 0.85 µg/l at admission and increased to 10.6 µg/l at discharge. After 2.5 years, the patient’s weight was 49 kg yielding in a BMI of 18.29 kg/m^2^, while leptin levels were 6.61 µg/l. One in vitro study validated the functional impact of the truncated protein reporting that this variant caused a complete loss of function^47^.

The other two non-synonymous and rare variants rs121913557 (p.Val50Leu) and rs13447329 (p.Thr112Met) were detected in both study groups. While in silico analyses hinted at a putative pathogenic and protein destabilizing impact of rs121913557, no functional implications for rs13447329 were detected (see Table 2). Previous studies analysing rs13447329 effects in vitro resulted in ambiguous findings. Thus, increased agonist potency and affinity for α-MSH were detected^53,54^. The mutant receptor further showed a decrease in cell surface expression in comparison to the WT^53^. Yet, multiple studies reported no functional difference of the mutant MC4R caused by this variant and the WT receptor^48,51,55^. We could not detect any studies in the evaluated literature resources, which have performed functional analyses for the Leu50 allele of rs121913557 so far (see Supplementary Tables 3, 4 and 5).

Both synonymous variants identified (p.Tyr153= and p.Val193=) were detected each in one proband (see Table 1). The variant p.Val193= was detected once in the lean control group and was implied to be benign (see Table 2). The other synonymous and novel variant p.Tyr153= was found in one patient with AN (BMI: 17.29 kg/m^2^). For this variant, in silico tools revealed a high capacity to affect splicing and to be pathogenic (see Table 2). Within a literature search, no previously published functional in vitro studies were available (see Supplementary Tables 3, 4 and 5).

## Discussion

Genetic variants in genes of the leptin-melanocortin system, like *LEP* and *MC4R*, are predominantly associated with monogenic forms of obesity^13,15,18,20,36,39,56–58^. Reports emerged indicating that some variants in these genes also predispose to a lower BMI^21,22,59^. Genetic correlations between BMI, obesity and AN have been uncovered^9,24–26^. In fact, genetic variants for AN are negatively correlated with BMI, leptin levels, body fat percentage, waist circumference, overweight as well as obesity^9,26^. Previously, we have identified nine SNPs at three independent genetic loci being associated with low BMI and AN^24^. To expand the findings of our previous mutation screens^36,39^, we have sequenced the coding regions of *LEP* and *MC4R* in 462 female patients with AN, and 445 healthy-lean controls. Collectively, we have detected four variants in the *LEP* gene and eight variants in *MC4R*. Strikingly, we detected known variants leading to reduced or even loss of function which are associated with obesity^39,60^. Yet, the *MC4R*-located variants rs2229616 (p.Val103Ile) and rs52820871 (p.Ile251Leu) detected in multiple patients with AN and healthy-lean controls were already known to be associated with a lower BMI and a reduced risk for obesity^21,22,44,61^. One GWAS for BMI performed for both sexes combined and separately revealed that rs2229616 (p.Val103Ile) was genome-wide significantly associated with the BMI in both sexes^38^. Thus confirming one of our previous studies^21^. In silico analyses indicated largely benign effects, while certain variants in both genes might impact the stability or torsion of the respective protein.

The relevance of the leptin-melanocortin system in body weight regulation, energy expenditure as well as AN is already known^10,29^. Patients with AN have reduced serum leptin levels, which typically rise as weight is restored^27–29^. Strikingly, low leptin levels were linked to AN hallmark characteristics, like hyperactivity and amenorrhea^62^. A recent Mendelian randomization study has reported that low leptin levels are correlated with a higher risk of AN. This correlation was also evident when analysing an exclusively female dataset. Notably, a correlation of AN with leptin levels was not detected^31^. Beyond, off-label treatment of patients with AN (case studies) with recombinant leptin helped to improve cognition, emotions, and behavioural traits, like hyperactivity and repetitive thoughts of food^32–34^. However, it remains uncertain how variants in the leptin gene have an effect on the etiology of AN. The here detected variant leading to the amino acid exchange of p.Val94Met was already linked to lower leptin levels^42^, which are known to increase AN risk^31^. Therefore, one could hypothesise that depending on other genetic variants or environmental exposures, one person may develop AN while another develops obesity. Yet, if this variant had an effect on body weight or reduced body weight, we would expect it to be equally present in underweight but healthy controls and not to be associated with obesity. Given that AN and obesity share some comorbidities, such as major depressive disorder^63–65^ as well as body dissatisfaction^1,66,67^ and regarding that metreleptin treatment in patients with AN and in one female with congenital leptin deficiency had beneficial effects on the mental traits^32–34,68^, the *LEP* variant might impact mental factors relevant for both, AN and obesity. Yet, further research is needed.

Evidence for the involvement of leptin’s downstream target *MC4R* in AN is mostly based on animal models. In fact, the activation of *MC4R* in rats decreased food intake, while coincidently activating the hypothalamic–pituitary–adrenal axis. This activation eventually led to increased motor activity. It was suggested that genetic variants might trigger this coherence and thus lead to a prolonged stimulation of the leptin-melanocortin-system supporting the development of AN^5^. Yet, especially as overlaps between genetic loci relevant for low BMI and AN are known^24,25^, it is feasible that genetic variants in genes of the leptin-melanocortin system, impact the etiology of AN.

However, to date, no significant associations of genetic variants in these genes with AN were reported^36,37^. Due to our still limited sample size and generally low allele frequencies of the here detected variants, we were also unable to report associations with AN. Nevertheless, this study was able to expand our previously published mutation screens for both genes^36,39^. Our extended sample size enabled us to discover new variants in *LEP* and *MC4R*, which potentially are relevant for AN. This emphasises the ongoing necessity for comprehensive large-scale genetic studies. Here, we did detect variants in *LEP* and *MC4R*, which were previously described to cause a partial or even complete loss of function in humans when present in a homozygous state and were thus associated with severe forms of obesity^39,51,60^. For instance, in 1999, we have described the frameshift mutation (rs13447338) yielding in a truncated MC4R in one adolescent and her mother with severe obesity^39^. In the present study, we have detected the same frameshift variant in the heterozygous state in one patient with AN. Yet, as no premorbid weight data was available, it is feasible that this patient had premorbid obesity triggering the development of AN. Generally, 2–5% of obesity cases are attributable to *MC4R* variants^13^. For the here commonly detected *MC4R* variants, like p.Val103Ile which was detected in 2% of patients with AN and 1.5% of controls, similar or slightly lower frequencies have been reported in patients with obesity^21,61,69,70^. Interestingly, this frequency is lower in females with obesity than in males with obesity^61^. The p.Val103Ile comprises the first polygenic variant for body weight regulation. The infrequent allele is associated with a lower BMI^21^. Further, we have to note that pathogenic variants typically are present homozygously^15,71^. For heterozygous *MC4R* variants, carrier develop obesity later in childhood^13^, while for heterozygous *LEP* variants, carriers are mainly unaffected^15,71^. As we have exclusively detected heterozygous variants in both genes, the relevance remains unknown. Yet, one patient with AN was detected to harbour two variants simultaneously (rs2229616, p.Val103Ile and rs13447331, p.Ser127Leu). Leptin levels at admission were in the expected range^72^ and increased during in-patient treatment. Yet, after 2.5 years, leptin levels were rather high (43.31 µg/l). The variant leading to p.Val103Ile is known to lead to an elevated function^21,22,46^, while the amino acid substitution of serine to leucine at the 127^th^ position was described to lead to a LoF in the majority of parameters analysed^45,55,73^ (see Supplementary Tables 3, 4 and 5). Notably, certain studies also indicated no functional deviations for mutant receptors with either Ile103 or Leu127^48,49,54^. Yet, functional studies for a double mutant MC4R showed for example a reduced efficacy and potency^51^. Previously, we have also detected weight reducing variants in *MC4R* in patients with bulimia nervosa^74^. Further, the *FTO* variant rs9939609 associated with obesity^75,76^ was previously identified in patients with eating disorders^77,78^. Besides a nominal association with bulimia nervosa and anorexia nervosa^77^, a synergic effect of this variant on leptin levels with an another variant in *ABCA1* was described^78^.

In addition, a number of studies demonstrated that two types of *MC4R* variants exist which can be distinguished according to their distinct signalling behaviour^22,46^. For example, variants such as the rs13447329 (p.Thr112Met) detected here, are characterised by a LoF. Other variants, such as rs2229616 (p.Val103Ile), show a GoF phenotype^22,46^. The latter GoF variants are associated with a reduced risk for obesity and a lower BMI^21,22,44^. In fact, heterozygous carriers of GoF variants weighed on overage 0.39 kg/m^2^ less than non-carriers, while homozygotes even showed an average 0.88 kg/m^2^ decreased BMI^22^. Similarly, an animal model with transgenic mice expressing either Val103Ile or Ile251Leu exhibited GoF phenotypes. This was even more pronounced in female than male mice. For instance, female mice carrying the variant Val103Ile homozygous had a 40% lower abdominal white adipose tissue mass than their wildtype littermates. This difference was not detected in the male transgenic mice^23^.

Based on our data, we are unable to deduce a mechanism explaining how these variants may have a relevance for the phenotypes at the extremes of the weight scale. Yet, a resembling mechanism of action, as *FTO*’s rs9939609 is feasible^78^. Functional in vitro and in vivo studies under fasting and starvation conditions are needed to expand the findings of the general in vitro data already published for each variant.

We are aware that in silico tools have their limitations and can vary substantially in their specificity^79–81^. Particularly, predicting functional implications of GoF variants was found to be more challenging by various in silico tools than finding an accurate implication for a LoF variant^82^. Hence, we have additionally checked the already published in vitro and in vivo data stated in LitVar^2^ and on the www.mc4r.org.uk website. Nevertheless, no clear-cut results were obtained for most of the variants (see Supplementary Tables 2, 3, 4 and 5). Presumably, this is due to variations in study design as well as the time period in which the studies were conducted. For example, older studies increasingly failed to detect any differences between WT and mutant MC4R. This was particularly evident for studies investigating the common GoF variant rs2229616 (p.Val103Ile) in *MC4R*. Supposedly, this results from the technical advances and new experimental techniques implemented in recent years. In addition, there are many variants for which no or only very few functional studies have been completed. For example, only 16 studies have been conducted on the rs17151919 variant in the leptin gene, of which just one included functional in vitro analyses (see Supplementary Table 2). Conversely, for the common variant rs2229616 in *MC4R*, there are over 150 studies, of which numerous also present in vitro or even in vivo data (see Supplementary Tables 3, 4 and 5).

Given the reported genetic overlaps between BMI and AN^9,24–26^ and GoF variants in *LEP* and *MC4R* being associated with a lower BMI and a reduced odds for obesity^21–23,59^, we have Sanger sequenced the coding regions of *LEP* and *MC4R* in 462 female patients with AN and 445 healthy-lean controls. We have detected four variants in *LEP* and eight variants in the coding sequence of *MC4R*. Here we detected variants with a partial or complete LoF in vitro in heterozygous patients with AN. Homozygous carriers of these variants typically develop obesity.

## Methods

### Study groups

To detect variants in the coding regions of *LEP* and *MC4R*, 462 female patients with AN (acutely ill or recovered; age: 20.83 ± 8.63 years old; BMI: 16.48 ± 2.77 kg/m^2^; BMI-SDS_LMS_: − 2.55 ± 1.58; see Table 3) and 445 healthy-lean individuals without a diagnosed eating disorder (age: 26.04 ± 5.77 years old; BMI: 18.08 ± 1.13 kg/m^2^; BMI-SDS_LMS_: − 2.77 ± 0.58; see Table 3) were included. In previous studies we have already screened 49 patients with AN for *LEP* and 51 patients with AN and 25 lean individuals for *MC4R*^36,39^. Written informed consent was given by all participants and in case of minors by their parents. This study was approved by the Ethics committee of the respective Universities and was performed in accordance with the *Declaration of Helsinki*.

**Table 3:** Phenotypic characteristics of the investigated study groups.

Sample	Status	n	% female	Age in years	BMI in kg/m^2^	BMI-SDS_LMS_
Patients with AN	Total cases	462	100	20.83 (8.63)	16.48 (2.77)	− 2.55 (1.58)
	Acutely ill	368	100	19.31 (7.70)	15.43 (1.67)	− 2.99 (1.40)
	Recovered	94	100	26.76 (9.42)	20.57 (2.40)	− 0.82 (0.90)
Healthy-lean individuals	Controls	445	61.7	26.04 (5.77)	18.08 (1.13)	− 2.77 (0.58)

All data is represented as mean (SD).

BMI, body mass index; BMI-SDS_LMS_, body mass index standard deviation score; SD, standard deviation.

### Mutation screen

The coding regions of *LEP* (chr7: 128,241,278–128,257,629; GRCh38; ENSG00000174697) and *MC4R* (chr18: 60,371,062–60,372,775; GRCh38; ENSG00000166603) were Sanger sequenced in 462 patients with AN, and 445 healthy-lean individuals (see Table 3) to detect genetic variants. These were identified by performing polymerase chain reactions (PCR; Veriti 96-well Thermal Cycler, Applied Biosystems, Foster City, CA, USA) with coding sequence specific primers (see Supplementary Table 1). PCR products were sent for sequencing to MicroSynth Seqlab GmbH (Göttingen, Germany). At least two independent scientists performed the sequence analysis and genotype assignment using the SeqMan Pro software (v.11.0.0, DNAstar Inc., Madison, WI, USA). Discrepancies were solved by either reaching consensus or by re-sequencing.

### Hardy–Weinberg-Equilibrium

All detected variants were checked for compliance with the Hardy–Weinberg-Equilibrium (HWE). All variants fulfilled the HWE.

### In silico analyses and assessment of functional implications by published data

To uncover putative functional implications of the detected variants, in silico analyses applying various tools ensued. The detected variants were analysed pertaining their potential to alter splice sites (ESEfinder^83^) and their general pathogenicity (MutationTaster2021^40^; PredictSNP2^41^). Effects of non-synonymous variants on the protein structure of LEP or MC4R were examined using PANTHER-PSEP^84^, the Cologne University Protein Stability Analysis Tool^85^ (CUPSAT) and the tool Sorting from Intolerant to Tolerant^86^ (SIFT). The required protein structures were extracted from the Research Collaboratory for Structural Bioinformatics protein data bank (RCSB PDB): LEP (1AX8^87^) and MC4R (7AUE^88^).

To extend the functional implications derived from the in silico analyses with previously published in vitro and in vivo data, the LitVar^2^^89^ (accessed on Sep. 26th 2022) database was screened for each variant in *LEP* and *MC4R* detected in this study. For *MC4R* variants, the website www.mc4r.org.uk (accessed on Sep. 26th 2022) summarizing publications for each variant, was additionally analysed. This website is provided by the University of Cambridge Metabolic Research Laboratories. A complete list of all studies provided by LitVar^2^ and www.mc4r.org.uk can be found in the Supplementary Table 5.

### Supplementary Information

Supplementary Information.

## Data Availability

The generated and analysed Sanger-sequencing data are available from the corresponding author on reasonable request. Data which was extracted from public websites or databases are included in the Supplementary Information files.
