# Supplementary material for: Unexpected identification of obesity-associated mutations in LEP and MC4R genes in patients with anorexia nervosa
Source: Sci Rep. 2024 Mar 25;14:7067. doi: 10.1038/s41598-024-57517-w (PMC10963783; doi:10.1038/s41598-024-57517-w)
Supplement: Supplementary file 1 — Supplementary Information. [file 41598_2024_57517_MOESM1_ESM.pdf]

Supplementary Material

Unexpected identification of obesity-associated mutations in *LEP* and *MC4R* genes in patients with anorexia nervosa

Luisa Sophie Rajcsanyi, Yiran Zheng, Beate Herpertz-Dahlmann, Jochen Seitz, Martina de Zwaan, Wolfgang Herzog, Stefan Ehrlich, Stephan Zipfel, Katrin Giel, Karin Egberts, Roland Burghardt, Manuel Föcker, Jochen Antel, Pamela Fischer-Posovszky, Johannes Hebebrand and Anke Hinney

Supplementary Table 1: List of used primers

Gene	Fragment	Forward primer	Reverse primer	Fragment size (bp)
LEP	Exon 1	TCATCCCCGTCTGGTAATGT	CTATCTGGGTCCAGTGCCACT	342
	Exon 2	GATTCCTCCCACATGCTGAG	TGCAATGCTCTTCAATCCTG	496
MC4R	Exon 1	ACTGAGACGACTCCCTGAC	TGCAGAAGTACAATATTCAGG	1146

Supplementary Table 2: Previously published *in vitro* studies for the detected *LEP* variants.

Variant	Amino acid exchange	Data extracted from…	Number of studies found*	*In vitro* data available?	Study by	PMID	Implication
rs201523306	p.Cys7=	LitVar²	0	No	*NA*	*NA*	*NA*
rs13306517	p.Gln25=	LitVar²	13	No	*NA*	*NA*	*NA*
no rsID g.128254532C/T	p.Ser91=	LitVar²	1	No	*NA*	*NA*	*NA*
rs17151919	p.Val94Met	LitVar²	16	Yes	Yaghootkar et al. 2020	32917775	Yes

All previously published data regarding the here detected variants located in *LEP* were extracted from LitVar² ([1]). The number of studies reported by LitVar² solely represents all studies found by the tool. This does not imply that the relevant variant, allele, consequence or phenotype was referred to in the respective publications (*). For certain variants no data was available (*NA*). An implication was present if differences between the wildtype and mutant protein were present in at least one characteristics or analysis. The complete output of LitVar² can be found in Supplementary Table 4. NA: not available. PMID: PubMedID.

Supplementary Table 3: Previously published *in vitro* studies for the detected *MC4R* variants.

Variant	Amino acid exchange	Data extracted from…	Number of studies found*	*In vitro* or *in vivo* data available?	Study by	PMID	(Functional) implication?
rs121913557	p.Val50Leu	LitVar^{2}	16	No	*NA*	*NA*	*NA*
		mc4r.org.uk	6	No	*NA*	*NA*	*NA*
rs2229616	p.Val103Ile	LitVar^{2}	154	Yes	[2]	33761344	Yes
					[3]	32059383	Yes
					[4]	31002796	Yes
					[5]	32916307	Yes
					[6]	14973783	No
					[7]	24385306	Yes (if double mutant with Ser127Leu)
					[8]	16886960	No
					[9]	23146882	No (if single mutant) Yes (if double mutant with Ser127Leu)
		mc4r.org.uk	14	Yes	[10]	10078568	No
					[11]	10585465	No
					[12]	12970296	No
					[13]	16752916	Yes
					[14]	22106157	No
					[9]	23146882[a]	No (if single mutant) Yes (if double mutant with Ser127Leu)
					[7]	24385306[a]	Yes (if double mutant with Ser127Leu)
					[2]	33761344[a]	Yes

rs13447329	p.Thr112Met	LitVar²	31	Yes	[9]	23146882	No
					[15]	25332687	Yes
					[16]	16030156	No
					[17]	14764818	No
		mc4r.org.uk	22	Yes	[10]	10078568	No
					[13]	16752916	Yes
					[9]	23146882[a]	No
					[18]	12690102	Yes
					[12]	12970296	no
					[17]	14764818[a]	No
rs13447331	p.Ser127Leu	LitVar²	40	Yes	[3]	32059383	Yes
					[7]	24385306	Yes
					[17]	14764818	Yes
					[19]	19298524	Yes
					[20]	30048591	Yes
					[21]	17668051	Yes
					[22]	23791567	Yes
					[23]	17590021	Yes
		mc4r.org.uk	15	Yes	[24]	12499395	Yes
					[21]	17668051[a]	Yes
					[7]	24385306[a]	Yes
					[9]	23146882	Yes (for double mutants)
					[12]	12970296	Yes
					[13]	16752916	No
					[19]	19298524[a]	Yes
					[17]	14764818	No
novel variant	p.Tyr153Tyr	LitVar²	0	No	*NA*	*NA*	*NA*
		mc4r.org.uk	1	No	*NA*	*NA*	*NA*
no rsID	p.Val193Val	LitVar²	1	No	*NA*	*NA*	*NA*
		mc4r.org.uk	1	No	*NA*	*NA*	*NA*
rs13447338	p.Leu211fsX	LitVar²	2	Yes	[12]	12970296	Yes
		mc4r.org.uk	1	No	*NA*	*NA*	*NA*
rs52820871	p.Ile251Leu	LitVar²	85	Yes	[2]	33761344	Yes

					[4]	31002796	Yes
					[9]	23146882	No
					[25]	23251400	Yes
					[26]	16553946	No
					[7]	24385306	No
		mc4r.org.uk	17	Yes	[12]	12970296	No
					[13]	16752916	Yes
					[14]	22106157	No
					[9]	23146882[a]	No
					[2]	33761344[a]	Yes

All previously published studies regarding the here detected variants located in *MC4R* were either extracted from LitVar² ([1]) or the mc4r.org.uk website. The number of studies reported solely represents all studies found by the tools/website. It does not imply that the relevant variant, allele, consequence or phenotype was referred to in the respective publications (*). A functional implication was given if at least one of parameters analysed in the studies was altered. Overlaps between the output of LitVar² and the website are possible and marked with [a]. For certain variants no data was available (*NA*). The complete output of LitVar² and www.mc4r.org.uk can be found in Supplementary Table 4. NA: not available. PMID: PubMedID.

Supplementary Table 4: Output of LitVar².

Gene	Variant	Number of studies found	PMID	Title
LEP	rs201523306	0	-	-
	rs133065017	13	33941261	Association of apolipoprotein B XbaI (rs693) polymorphism and gallstone disease risk based on a comprehensive analysis
			33233816	The Genetic Basis of Obesity and Related Metabolic Diseases in Humans and Companion Animals
			30467071	Role of rs670 variant of APOA1 gene on metabolic response after a high fat vs. a low fat hypocaloric diets in obese human subjects.
			29974678	A likely pathogenic variant putatively affecting splicing of PIGA identified in a multiple congenital anomalies hypotonia-seizures syndrome 2 (MCAHS2) family pedigree via whole-exome sequencing
			28953935	Association of MTTP gene variants with pediatric NAFLD: A candidate-gene-based analysis of single nucleotide variations in obese children
			28404951	Search for rare protein altering variants influencing susceptibility to multiple myeloma
			26523220	Correlational studies on insulin resistance and leptin gene polymorphisms in peritoneal dialysis patients
			25668207	Genetic Investigation of Bisphosphonate-Related Osteonecrosis of Jaw (BRONJ) via Whole Exome Sequencing and Bioinformatics
			25310821	A Genome-Wide Association Study Identifies Potential Susceptibility Loci for Hirschsprung Disease
			24219164	Effect of a poly(ADP-ribose) polymerase-1 inhibitor against esophageal squamous cell carcinoma cell lines
			23026132	Resistance to antidepressant treatment is associated with polymorphisms in the leptin gene, decreased leptin mRNA expression, and decreased leptin serum levels
			20232565	Identification of leptin gene variants in school children with early onset obesity.
			18997673	Functional consequences of the human leptin receptor (LEPR) Q223R transversion

	no rsID (p.Ser91Ser)	1	9857981	No evidence for involvement of the leptin gene in anorexia nervosa, bulimia nervosa, underweight or early onset extreme obesity: identification of two novel mutations in the coding sequence and a novel polymorphism in the leptin gene linked upstream region.
	rs17151919	16	34642196	Infant feeding, appetite and satiety regulation, and adiposity during infancy: a study design and protocol of the 'MAS-Lactancia' birth cohort
			34594363	Suggestive Evidence for Causal Effect of Leptin Levels on Risk for Anorexia Nervosa: Results of a Mendelian Randomization Study
			33631239	ADIPOQ and LEP variants on asthma and atopy: Genetic association modified by overweight.
			33561332	Associations between gene polymorphisms and selected meat traits in cattle : A review
			32917775	Genetic Studies of Leptin Concentrations Implicate Leptin in the Regulation of Early Adiposity.
			30937429	An enhanced workflow for variant interpretation in UniProtKB/Swiss-Prot improves consistency and reuse in ClinVar
			28542631	Concordance of bioactive vs. total immunoreactive serum leptin levels in children with severe early onset obesity
			28436986	Adiposity Amplifies the Genetic Risk of Fatty Liver Disease Conferred by Multiple Loci
			26659599	Reproducible Analysis of Post-Translational Modifications in Proteomes:Application to Human Mutations
			24349080	Mechanistic Phenotypes: An Aggregative Phenotyping Strategy to Identify Disease Mechanisms Using GWAS Data
			23760429	Association between polymorphisms in leptin, leptin receptor, and beta-adrenergic receptor genes and bone mineral density in postmenopausal Korean women.
			23033454	Variation in genes related to obesity, weight and weight change and risk of contralateral breast cancer in the WECARE Study population
			23026132	Resistance to antidepressant treatment is associated with polymorphisms in the leptin gene, decreased leptin mRNA expression, and decreased leptin serum levels
			22614171	Appetite regulation genes are associated with body mass index in black South African adolescents: a genetic association study

			21773001	Variations in Adipokine Genes AdipoQ, Lep, and LepR are Associated with Risk for Obesity-Related Metabolic Disease: The Modulatory Role of Gene-Nutrient Interactions
			20642810	Candidate Genes from Molecular Pathways Related to Appetite Regulatory Neural Network and Adipocyte Homeostasis and Obesity: the Coronary Artery Risk Development in Young Adults (CARDIA) Study
MC4R	rs121913557	16	32952152	Evaluation of the MC4R gene across eMERGE network identifies many unreported obesity-associated variants
			31855179	Common activation mechanism of class A GPCRs
			31358043	A validated single-cell-based strategy to identify diagnostic and therapeutic targets in complex diseases
			30665703	Assessing the Pathogenicity, Penetrance, and Expressivity of Putative Disease-Causing Variants in a Population Setting
			29031731	Evaluation of a melanocortin-4 receptor (MC4R) agonist (Setmelanotide) in MC4R deficiency
			28433713	Biased Signaling at Neural Melanocortin Receptors in Regulation of Energy Homeostasis
			26986070	Characterization of Disease-Associated Mutations in Human Transmembrane Proteins
			26229975	How genetic errors in GPCRs affect their function: Possible therapeutic strategies
			26167768	An siRNA-based functional genomics screen for the identification of regulators of ciliogenesis and ciliopathy genes
			26147798	The Use of Non-Variant Sites to Improve the Clinical Assessment of Whole-Genome Sequence Data
			25332687	Defect in MAPK Signaling As a Cause for Monogenic Obesity Caused By Inactivating Mutations in the Melanocortin-4 Receptor Gene
			22817722	Pharmacological chaperones increase the cell-surface expression of intracellularly retained mutants of the melanocortin 4 receptor with unique rescuing efficacy profiles.
			20462274	Pharmacological Characterization of 30 Human Melanocortin-4 Receptor Polymorphisms with the Endogenous Proopiomelanocortin Derived Agonists, Synthetic Agonists, and the Endogenous Agouti-Related Protein (AGRP) Antagonist

			19417090	In silico mutagenesis: a case study of the melanocortin 4 receptor
			12959994	Functional characterization of melanocortin-4 receptor mutations associated with childhood obesity.
			11487744	Mutational analysis of melanocortin-4 receptor, agouti-related protein, and alpha-melanocyte-stimulating hormone genes in severely obese children.
	rs2229616	154	9267995	Molecular screening of the human melanocortin-4 receptor gene: identification of a missense variant showing no association with obesity, plasma glucose, or insulin.
			35095762	Weight and Glycemic Control Outcomes of Bariatric Surgery and Pharmacotherapy in Patients With Melanocortin-4 Receptor Deficiency
			34900785	Effect of dietary fat intake and genetic risk on glucose and insulin-related traits in Brazilian young adults
			34683180	A Clinical-Genetic Score for Predicting Weight Loss after Bariatric Surgery: The OBEGEN Study
			34414523	FTO-rs9939609 Polymorphism is a Predictor of Future Type 2 Diabetes: A Population-Based Prospective Study
			34315947	Association of CNR1 and INSIG2 polymorphisms with antipsychotics-induced weight gain: a prospective nested case-control study
			34045736	Loss of function mutations in the melanocortin 4 receptor in a UK birth cohort
			33807560	A Systematic Review of Genetic Polymorphisms Associated with Binge Eating Disorder.
			33761344	Human MC4R variants affect endocytosis, trafficking and dimerization revealing multiple cellular mechanisms involved in weight regulation
			33498618	Interaction between Vitamin D-Related Genetic Risk Score and Carbohydrate Intake on Body Fat Composition: A Study in Southeast Asian Minangkabau Women
			33362866	Identification of a Rare and Potential Pathogenic MC4R Variant in a Brazilian Patient With Adulthood-Onset Severe Obesity
			33233816	The Genetic Basis of Obesity and Related Metabolic Diseases in Humans and Companion Animals
			33066636	Statistical and Machine-Learning Analyses in Nutritional Genomics Studies
			33045981	Interaction between the genetic risk score and dietary protein intake on cardiometabolic traits in Southeast Asian

			32952152	Evaluation of the MC4R gene across eMERGE network identifies many unreported obesity-associated variants
			32921795	Obesity treatment effect in Danish children and adolescents carrying Melanocortin-4 Receptor mutations
			32916307	Mouse models for V103I and I251L gain of function variants of the human MC4R display decreased adiposity but are not protected against a hypercaloric diet
			32665031	PWAS: proteome-wide association study:linking genes and phenotypes by functional variation in proteins
			32605047	Interaction between Metabolic Genetic Risk Score and Dietary Fatty Acid Intake on Central Obesity in a Ghanaian Population
			32316318	Metabolic Reprogramming in Health and Disease
			32185475	Melanocortin 4 receptor (MC4R) gene variants in children and adolescents having familial early-onset obesity: genetic and clinical characteristics
			32114639	Genetic determinants of gestational diabetes mellitus: a case-control study in two independent populations.
			32059383	Differential Signaling Profiles of MC4R Mutations with Three Different Ligands
			31947684	The Effect Sizes of PPARgamma rs1801282, FTO rs9939609, and MC4R rs2229616 Variants on Type 2 Diabetes Mellitus Risk among the Western Saudi Population: A Cross-Sectional Prospective Study
			31890664	A nutrigenetic approach for investigating the relationship between vitamin B12 status and metabolic traits in Indonesian women
			31492854	Components of genetic associations across 2,138 phenotypes in the UK Biobank highlight adipocyte biology
			31484069	Peptide/Receptor Co-evolution Explains the Lipolytic Function of the Neuropeptide TLQP-21
			31358043	A validated single-cell-based strategy to identify diagnostic and therapeutic targets in complex diseases
			31348498	Voice break in boys:temporal relations with other pubertal milestones and likely causal effects of BMI
			31263163	Genome-wide Association Study of Change in Fasting Glucose over time in 13,807 non-diabetic European Ancestry Individuals

			31178821	Association Between Premorbid Body Mass Index and Amyotrophic Lateral Sclerosis: Causal Inference Through Genetic Approaches
			31064983	Pancreatic islet chromatin accessibility and conformation reveals distal enhancer networks of type 2 diabetes risk
			31048771	Genetic Predisposition Impacts Clinical Changes in a Lifestyle Coaching Program
			31002796	Human Gain-of-Function MC4R Variants Show Signaling Bias and Protect against Obesity
			30981838	Genetic screening for MC4R gene identifies three novel mutations associated with severe familiar obesity in a cohort of Spanish individuals.
			30937429	An enhanced workflow for variant interpretation in UniProtKB/Swiss-Prot improves consistency and reuse in ClinVar
			30894629	A reference collection of patient-derived cell line and xenograft models of proneural, classical and mesenchymal glioblastoma
			30863132	Identification of the MC4R start lost mutation in a morbidly obese Brazilian patient
			30840888	APOBEC Mutagenesis and Copy-Number Alterations Are Drivers of Proteogenomic Tumor Evolution and Heterogeneity in Metastatic Thoracic Tumors
			30650367	Multiomic Profiling Identifies cis-Regulatory Networks Underlying Human Pancreatic beta Cell Identity and Function
			30300385	A type 2 diabetes disease module with a high collective influence for Cdk2 and PTPLAD1 is localized in endosomes
			30256453	Rare genetic variation in mitochondrial pathways influences the risk for Parkinson's disease
			30214008	Whole genome sequencing identifies high-impact variants in well-known pharmacogenomic genes
			30134862	Relevance of polymorphisms in MC4R and BDNF in short normal stature
			30121879	Genetics of Severe Obesity
			30068297	Heterozygous versus homozygous phenotype caused by the same MC4R mutation: novel mutation affecting a large consanguineous kindred
			30066661	An update on obesity research pattern among adults in Malaysia: a scoping review

			30032228	Associations between Single Nucleotide Polymorphisms and Total Energy, Carbohydrate, and Fat Intakes: A Systematic Review
			29970889	Elucidating the genetic basis of social interaction and isolation
			29966015	Computational analyses of obesity associated loci generated by genome-wide association studies
			29736163	Genetic Aspects of Obesity
			29679223	Association between LEPR, FTO, MC4R, and PPARG-2 polymorphisms with obesity traits and metabolic phenotypes in school-aged children
			29658973	Searching for ancient balanced polymorphisms shared between Neanderthals and Modern Humans
			28828082	Impact of the Polymorphism Near MC4R (rs17782313) on Obesity- and Metabolic-Related Traits in Women Participating in an Aerobic Training Program
			28757201	Continuity and Admixture in the Last Five Millennia of Levantine History from Ancient Canaanite and Present-Day Lebanese Genome Sequences
			28755272	Polymorphism and methylation of the MC4R gene in obese and non-obese dogs
			28567303	Whole-genome sequencing expands diagnostic utility and improves clinical management in paediatric medicine
			28440294	Next-generation DNA sequencing identifies novel gene variants and pathways involved in specific language impairment
			28391526	Trans-ethnic fine-mapping of genetic loci for body mass index in the diverse ancestral populations of the Population Architecture using Genomics and Epidemiology (PAGE) Study reveals evidence for multiple signals at established loci
			28105929	A compendium of human genes regulating feeding behavior and body weight, its functional characterization and identification of GWAS genes involved in brain-specific PPI network
			27575456	Multi-Variant Study of Obesity Risk Genes in African Americans: The Jackson Heart Study
			27270441	Molecular analysis of urothelial cancer cell lines for modeling tumor biology and drug response
			27013903	Genetics and Epigenetics of Eating Disorders

			26986070	Characterization of Disease-Associated Mutations in Human Transmembrane Proteins
			26828654	Analysis of Genes Involved in Body Weight Regulation by Targeted Re-Sequencing
			26788538	Allelic variants of the Melanocortin 4 receptor (MC4R) gene in a South African study group
			26659599	Reproducible Analysis of Post-Translational Modifications in Proteomes:Application to Human Mutations
			26600159	Genome-Wide Association Study Identifies That the ABO Blood Group System Influences Interleukin-10 Levels and the Risk of Clinical Events in Patients with Acute Coronary Syndrome
			26474449	Practical Experience of the Application of a Weighted Burden Test to Whole Exome Sequence Data for Obesity and Schizophrenia
			26244670	The prevalence of melanocortin-4 receptor gene mutations in Slovak obese children and adolescents.
			26229975	How genetic errors in GPCRs affect their function: Possible therapeutic strategies
			26167768	An siRNA-based functional genomics screen for the identification of regulators of ciliogenesis and ciliopathy genes
			25919555	[Mutation screening and function prediction of melanocortin-4 receptor gene in obese children].
			25905921	XomAnnotate: Analysis of Heterogeneous and Complex Exome- A Step towards Translational Medicine
			25668207	Genetic Investigation of Bisphosphonate-Related Osteonecrosis of Jaw (BRONJ) via Whole Exome Sequencing and Bioinformatics
			25579139	Genetic & epigenetic approach to human obesity
			25333361	Exome Sequencing Is an Efficient Tool for Variant Late-Infantile Neuronal Ceroid Lipofuscinosis Molecular Diagnosis
			25032700	NCI-60 Whole Exome Sequencing and Pharmacological CellMiner Analyses
			24820477	Genetic Association Study of Adiposity and Melanocortin-4 Receptor (MC4R) Common Variants: Replication and Functional Characterization of Non-Coding Regions

			24705671	Long-Term Weight-Loss in Gastric Bypass Patients Carrying Melanocortin 4 Receptor Variants
			24611737	Novel variants in the MC4R and LEPR genes among severely obese children from the Iberian population.
			24518831	Controversial association results for INSIG2 on body mass index may be explained by interactions with age and with MC4R.
			24385306	The role of common and rare MC4R variants and FTO polymorphisms in extreme form of obesity.
			24276017	Brain-Derived Neurotrophic Factor in Human Subjects with Function-Altering Melanocortin-4 Receptor Variants
			24267414	Impact of obesity-related genes in Spanish population
			23996627	Family of melanocortin receptor (MCR) genes in mammals:mutations, polymorphisms and phenotypic effects
			23819521	Assessment of computational methods for predicting the effects of missense mutations in human cancers
			23505181	The missense variation landscape of FTO, MC4R and TMEM18 in obese children of African ancestry
			23485949	Polymorphic variants of neurotransmitter receptor genes may affect sexual function in aging males: data from the HALS study.
			23270367	Mutation screen in the GWAS derived obesity gene SH2B1 including functional analyses of detected variants
			23185251	Melanocortin-4 Receptor Mutations and Polymorphisms Do Not Affect Weight Loss after Bariatric Surgery
			23101478	Single nucleotide polymorphisms (SNPs) involved in insulin resistance, weight regulation, lipid metabolism and inflammation in relation to metabolic syndrome: an epidemiological study
			23049848	Association between Common Polymorphism near the MC4R Gene and Obesity Risk: A Systematic Review and Meta-Analysis
			23033454	Variation in genes related to obesity, weight and weight change and risk of contralateral breast cancer in the WECARE Study population
			22989167	Association of melanocortin-4 receptor gene polymorphisms with obesity-related parameters in Malaysian Malays.

			22822657	Prevalence of melanocortin receptor 4 (MC4R) V103I gene variant and its association with obesity among the Kampar Health Clinic cohort, Perak, Malaysia.
			22535570	Is there a genetic cause of appetite loss?:an explorative study in 1,853 cancer patients
			22473907	P-selectin genotype is associated with the development of cancer cachexia
			22447289	Loss-of-function mutations in MC4R are very rare in the Greek severely obese adult population.
			22310352	Genetic association study between antipsychotic-induced weight gain and the melanocortin-4 receptor gene.
			22106157	Greater Impact of Melanocortin-4 Receptor Deficiency on Rates of Growth and Risk of Type 2 Diabetes During Childhood Compared With Adulthood in Pima Indians
			22043165	Genetics of Obesity: What have we Learned?
			22043164	Molecular Basis of Obesity: Current Status and Future Prospects
			22020349	Polymorphisms of INSIG2, MC4R, and LEP are associated with obesity- and metabolic-related traits in schizophrenic patients.
			21976721	The MC4R(I251L) allele is associated with better metabolic status and more weight loss after gastric bypass surgery.
			21934689	Is there a genetic cause for cancer cachexia? - a clinical validation study in 1797 patients
			21921652	Evaluation of the obesity genes FTO and MC4R and the type 2 diabetes mellitus gene TCF7L2 for contribution to stroke risk: The Mannheim-Heidelberg Stroke Study.
			21750520	Implication of European-derived adiposity loci in African Americans
			21404042	Missense mutations and polymorphisms of the MC4R gene in Polish obese children and adolescents in relation to the relative body mass index
			21295023	The genetic epidemiology of melanocortin 4 receptor variants.
			21085626	Investigation of a Genome Wide Association Signal for Obesity: Synthetic Association and Haplotype Analyses at the Melanocortin 4 Receptor Gene Locus
			20935630	Association analyses of 249,796 individuals reveal eighteen new loci associated with body mass index

			20682687	Common Variants in 40 Genes Assessed for Diabetes Incidence and Response to Metformin and Lifestyle Intervention in the Diabetes Prevention Program
			20587078	Genomic insights into early-onset obesity
			20530052	Melanocortin-4 receptor polymorphisms in Turkish pediatric obese patients.
			20462274	Pharmacological Characterization of 30 Human Melanocortin-4 Receptor Polymorphisms with the Endogenous Proopiomelanocortin Derived Agonists, Synthetic Agonists, and the Endogenous Agouti-Related Protein (AGRP) Antagonist
			20406574	Melanocortin-4 receptor gene variants in Chilean families: association with childhood obesity and eating behavior.
			20127379	From monogenic to polygenic obesity: recent advances
			20092643	Large effects on body mass index and insulin resistance of fat mass and obesity associated gene (FTO) variants in patients with polycystic ovary syndrome (PCOS)
			20054160	Polygenic obesity in humans.
			20033240	Eating disorders: the current status of molecular genetic research
			20004082	Update on Melanocortin Interventions for Cachexia: Progress Toward Clinical Application
			19696756	Association of the MC4R V103I polymorphism with obesity: a Chinese case-control study and meta-analysis in 55,195 individuals.
			19417090	In silico mutagenesis: a case study of the melanocortin 4 receptor
			19284607	Prevalence of pathogenetic MC4R mutations in Italian children with early Onset obesity, tall stature and familial history of obesity
			19073769	Variants Near MC4R Are Associated With Obesity and Influence Obesity-Related Quantitative Traits in a Population of Middle-Aged People: Studies of 14,940 Danes
			19046411	Obesity genes: so close and yet so far...
			18835933	Lower Metabolic Rate in Individuals Heterozygous for Either a Frameshift or a Functional Missense MC4R Variant
			18779298	Association of the melanocortin-4 receptor V103I polymorphism with dietary intake in severely obese individuals

			18559663	Prevalence of Melanocortin-4 Receptor Deficiency in Europeans and Their Age-Dependent Penetrance in Multigenerational Pedigrees
			18454148	Common variants near MC4R are associated with fat mass, weight and risk of obesity
			18377640	Val103Ile polymorphism of the melanocortin-4 receptor gene (MC4R) in cancer cachexia
			18239646	Association of the MC4R V103I polymorphism with the metabolic syndrome: the KORA Study.
			17587397	Genetic variation and decreased risk for obesity in the Atherosclerosis Risk in Communities Study.
			17519222	Non-synonymous polymorphisms in melanocortin-4 receptor protect against obesity: the two facets of a Janus obesity gene.
			17418015	[Association between melanocortin 4 receptor gene polymorphisms and obesity].
			17356525	The V103I polymorphism of the MC4R gene and obesity: population based studies and meta-analysis of 29 563 individuals
			17286227	A novel non-synonymous mutation in the melanocortin-4 receptor gene (MC4R) in a 2-year-old Austrian girl with extreme obesity.
			17196040	Unraveling the Genetics of Human Obesity
			17185898	Several mutations in the melanocortin 4 receptor gene are associated with obesity in Chinese children and adolescents.
			17072869	Prevalence and functionality of paucimorphic and private MC4R mutations in a large, unselected European British population, scanned by meltMADGE.
			16886960	Identification and functional characterization of three novel human melanocortin-4 receptor gene variants in an obese Chinese population.
			16278267	The 103I variant of the melanocortin 4 receptor is associated with low serum triglyceride levels.
			16231025	Screening for melanocortin-4 receptor mutations in a cohort of Belgian morbidly obese adults and children.
			15805150	Association of the 103I MC4R allele with decreased body mass in 7937 participants of two population based surveys.
			15292469	The Val103Ile polymorphism of melanocortin-4 receptor regulates energy expenditure and weight gain.

			15037865	Binge-eating episodes are not characteristic of carriers of melanocortin-4 receptor gene mutations.
			14973783	Melanocortin-4 receptor gene variant I103 is negatively associated with obesity.
			14764818	Identification and characterization of melanocortin-4 receptor gene mutations in morbidly obese finnish children and adults.
			14764812	Genetic screening for melanocortin-4 receptor mutations in a cohort of Italian obese patients: description and functional characterization of a novel mutation.
			12629567	A novel nonsense mutation in the melanocortin-4 receptor associated with obesity in a Spanish population.
			11692184	A missense mutation in the human melanocortin-4 receptor gene in relation to abdominal obesity and salivary cortisol.
			11246450	Molecular scanning for mutations in the melanocortin-4 receptor gene in obese/diabetic Japanese.
			10199800	Several mutations in the melanocortin-4 receptor gene including a nonsense and a frameshift mutation associated with dominantly inherited obesity in humans.
	rs13447329	31	34045736	Loss of function mutations in the melanocortin 4 receptor in a UK birth cohort
			33202557	The Melanocortin System behind the Dysfunctional Eating Behaviors
			32921795	Obesity treatment effect in Danish children and adolescents carrying Melanocortin-4 Receptor mutations
			32153512	Rare Variants in Genes Linked to Appetite Control and Hypothalamic Development in Early-Onset Severe Obesity
			31646011	The role of GPCRs in bone diseases and dysfunctions
			31088516	Identification of genetic association between cardiorespiratory fitness and the trainability genes in childhood acute lymphoblastic leukemia survivors
			30134862	Relevance of polymorphisms in MC4R and BDNF in short normal stature
			28839234	Identification of NCAN as a candidate gene for developmental dyslexia
			28433713	Biased Signaling at Neural Melanocortin Receptors in Regulation of Energy Homeostasis
			27706562	Prevalence of mutations in LEP, LEPR, and MC4R genes in individuals with severe obesity.

			26986070	Characterization of Disease-Associated Mutations in Human Transmembrane Proteins
			26659599	Reproducible Analysis of Post-Translational Modifications in Proteomes:Application to Human Mutations
			26229975	How genetic errors in GPCRs affect their function: Possible therapeutic strategies
			25332687	Defect in MAPK Signaling As a Cause for Monogenic Obesity Caused By Inactivating Mutations in the Melanocortin-4 Receptor Gene
			25188385	A Probabilistic Model to Predict Clinical Phenotypic Traits from Genome Sequencing
			25032700	NCI-60 Whole Exome Sequencing and Pharmacological CellMiner Analyses
			24705671	Long-Term Weight-Loss in Gastric Bypass Patients Carrying Melanocortin 4 Receptor Variants
			23819521	Assessment of computational methods for predicting the effects of missense mutations in human cancers
			23146882	Clinical and functional relevance of melanocortin-4 receptor variants in obese German children.
			22447289	Loss-of-function mutations in MC4R are very rare in the Greek severely obese adult population.
			21404042	Missense mutations and polymorphisms of the MC4R gene in Polish obese children and adolescents in relation to the relative body mass index
			21085626	Investigation of a Genome Wide Association Signal for Obesity: Synthetic Association and Haplotype Analyses at the Melanocortin 4 Receptor Gene Locus
			19417090	In silico mutagenesis: a case study of the melanocortin 4 receptor
			18974781	Cataloging Coding Sequence Variations in Human Genome Databases
			18559663	Prevalence of Melanocortin-4 Receptor Deficiency in Europeans and Their Age-Dependent Penetrance in Multigenerational Pedigrees
			17072869	Prevalence and functionality of paucimorphic and private MC4R mutations in a large, unselected European British population, scanned by meltMADGE.
			16231025	Screening for melanocortin-4 receptor mutations in a cohort of Belgian morbidly obese adults and children.

			16030156	Functional analyses of melanocortin-4 receptor mutations identified from patients with binge eating disorder and nonobese or obese subjects.
			14764818	Identification and characterization of melanocortin-4 receptor gene mutations in morbidly obese finnish children and adults.
			10903343	Dominant and recessive inheritance of morbid obesity associated with melanocortin 4 receptor deficiency.
			10078568	Identification and functional analysis of novel human melanocortin-4 receptor variants.
	rs13447331	40	34045736	Loss of function mutations in the melanocortin 4 receptor in a UK birth cohort
			32952152	Evaluation of the MC4R gene across eMERGE network identifies many unreported obesity-associated variants
			32384097	Identifying underlying medical causes of pediatric obesity: Results of a systematic diagnostic approach in a pediatric obesity center
			32316318	Metabolic Reprogramming in Health and Disease
			32059383	Differential Signaling Profiles of MC4R Mutations with Three Different Ligands
			31855179	Common activation mechanism of class A GPCRs
			31358043	A validated single-cell-based strategy to identify diagnostic and therapeutic targets in complex diseases
			30926952	Heterozygous rare genetic variants in non-syndromic early-onset obesity
			30048591	Insights into the Allosteric Mechanism of Setmelanotide (RM-493) as a Potent and First-in-Class Melanocortin-4 Receptor (MC4R) Agonist To Treat Rare Genetic Disorders of Obesity through an in Silico Approach
			30019023	Structural Biology Helps Interpret Variants of Uncertain Significance in Genes Causing Endocrine and Metabolic Disorders
			29273807	Protein-altering variants associated with body mass index implicate pathways that control energy intake and expenditure underpinning obesity
			28719003	Mutation intolerant genes and targets of FMRP are enriched for nonsynonymous alleles in schizophrenia
			28404951	Search for rare protein altering variants influencing susceptibility to multiple myeloma
			26986070	Characterization of Disease-Associated Mutations in Human Transmembrane Proteins

			26740555	Exome arrays capture polygenic rare variant contributions to schizophrenia
			26659599	Reproducible Analysis of Post-Translational Modifications in Proteomes:Application to Human Mutations
			26244670	The prevalence of melanocortin-4 receptor gene mutations in Slovak obese children and adolescents.
			26238496	Age of obesity onset in MC4R mutation carriers.
			26229975	How genetic errors in GPCRs affect their function: Possible therapeutic strategies
			26167768	An siRNA-based functional genomics screen for the identification of regulators of ciliogenesis and ciliopathy genes
			26147798	The Use of Non-Variant Sites to Improve the Clinical Assessment of Whole-Genome Sequence Data
			26047380	Melanocortin-4 receptor gene mutations in obese Slovak children.
			24611737	Novel variants in the MC4R and LEPR genes among severely obese children from the Iberian population.
			24385306	The role of common and rare MC4R variants and FTO polymorphisms in extreme form of obesity.
			23819521	Assessment of computational methods for predicting the effects of missense mutations in human cancers
			23791567	Activation of MAPK by inverse agonists in six naturally occurring constitutively active mutant human melanocortin-4 receptors.
			22447289	Loss-of-function mutations in MC4R are very rare in the Greek severely obese adult population.
			21404042	Missense mutations and polymorphisms of the MC4R gene in Polish obese children and adolescents in relation to the relative body mass index
			21341504	[Genetic variations in energy balance regulation].
			21085626	Investigation of a Genome Wide Association Signal for Obesity: Synthetic Association and Haplotype Analyses at the Melanocortin 4 Receptor Gene Locus
			20507906	PhenoHM: human-mouse comparative phenome-genome server
			19298524	Functional characterization and pharmacological rescue of melanocortin-4 receptor mutations identified from obese patients
			19284607	Prevalence of pathogenetic MC4R mutations in Italian children with early Onset obesity, tall stature and familial history of obesity

			19139070	Genome-wide analysis to predict protein sequence variations that change phosphorylation sites or their corresponding kinases
			18974781	Cataloging Coding Sequence Variations in Human Genome Databases
			18559663	Prevalence of Melanocortin-4 Receptor Deficiency in Europeans and Their Age-Dependent Penetrance in Multigenerational Pedigrees
			17668051	Engineering the Melanocortin-4 Receptor to Control Constitutive and Ligand-Mediated Gs Signaling In Vivo
			17590021	Peptide and small molecules rescue the functional activity and agonist potency of dysfunctional human melanocortin-4 receptor polymorphisms.
			17579204	Melanocortin 4 receptor mutations in obese Czech children: studies of prevalence, phenotype development, weight reduction response, and functional analysis.
			14764818	Identification and characterization of melanocortin-4 receptor gene mutations in morbidly obese finnish children and adults.
	novel (p.Tyr153Tyr)	0	-	-
	no rsID (p.Val193Val)	1	10199800	Several mutations in the melanocortin-4 receptor gene including a nonsense and a frameshift mutation associated with dominantly inherited obesity in humans.
	rs13447338	2	33362866	Identification of a Rare and Potential Pathogenic MC4R Variant in a Brazilian Patient With Adulthood-Onset Severe Obesity
			17941900	Novel melanocortin 4 receptor gene mutations in severely obese children.
	rs52820871	85	35328759	Neurohormonal Changes in the Gut-Brain Axis and Underlying Neuroendocrine Mechanisms following Bariatric Surgery
			34887725	Role of the Autonomic Nervous System in Mechanism of Energy and Glucose Regulation Post Bariatric Surgery
			34683180	A Clinical-Genetic Score for Predicting Weight Loss after Bariatric Surgery: The OBEGEN Study
			34045736	Loss of function mutations in the melanocortin 4 receptor in a UK birth cohort
			33807560	A Systematic Review of Genetic Polymorphisms Associated with Binge Eating Disorder.
			33761344	Human MC4R variants affect endocytosis, trafficking and dimerization revealing multiple cellular mechanisms involved in weight regulation

			33362866	Identification of a Rare and Potential Pathogenic MC4R Variant in a Brazilian Patient With Adulthood-Onset Severe Obesity
			32952152	Evaluation of the MC4R gene across eMERGE network identifies many unreported obesity-associated variants
			32921795	Obesity treatment effect in Danish children and adolescents carrying Melanocortin-4 Receptor mutations
			32916307	Mouse models for V103I and I251L gain of function variants of the human MC4R display decreased adiposity but are not protected against a hypercaloric diet
			32697988	Human Labor Pain Is Influenced by the Voltage-Gated Potassium Channel KV6.4 Subunit
			32665031	PWAS: proteome-wide association study:linking genes and phenotypes by functional variation in proteins
			31855179	Common activation mechanism of class A GPCRs
			31597922	Distinctive mutational spectrum and karyotype disruption in long-term cisplatin-treated urothelial carcinoma cell lines
			31358043	A validated single-cell-based strategy to identify diagnostic and therapeutic targets in complex diseases
			31064983	Pancreatic islet chromatin accessibility and conformation reveals distal enhancer networks of type 2 diabetes risk
			31035493	Melanocortin-4 Receptor and Lipocalin 2 Gene Variants in Spanish Children with Abdominal Obesity: Effects on BMI-SDS after a Lifestyle Intervention
			31002796	Human Gain-of-Function MC4R Variants Show Signaling Bias and Protect against Obesity
			30981838	Genetic screening for MC4R gene identifies three novel mutations associated with severe familiar obesity in a cohort of Spanish individuals.
			30863132	Identification of the MC4R start lost mutation in a morbidly obese Brazilian patient
			30256453	Rare genetic variation in mitochondrial pathways influences the risk for Parkinson's disease
			30134862	Relevance of polymorphisms in MC4R and BDNF in short normal stature
			30121879	Genetics of Severe Obesity
			30068297	Heterozygous versus homozygous phenotype caused by the same MC4R mutation: novel mutation affecting a large consanguineous kindred

			30019023	Structural Biology Helps Interpret Variants of Uncertain Significance in Genes Causing Endocrine and Metabolic Disorders
			29273807	Protein-altering variants associated with body mass index implicate pathways that control energy intake and expenditure underpinning obesity
			29230297	From genetics and epigenetics to the future of precision treatment for obesity
			28755272	Polymorphism and methylation of the MC4R gene in obese and non-obese dogs
			28567303	Whole-genome sequencing expands diagnostic utility and improves clinical management in paediatric medicine
			27460824	The impact of tumor profiling approaches and genomic data strategies for cancer precision medicine
			27013903	Genetics and Epigenetics of Eating Disorders
			26986070	Characterization of Disease-Associated Mutations in Human Transmembrane Proteins
			26828654	Analysis of Genes Involved in Body Weight Regulation by Targeted Re-Sequencing
			26788538	Allelic variants of the Melanocortin 4 receptor (MC4R) gene in a South African study group
			26659599	Reproducible Analysis of Post-Translational Modifications in Proteomes:Application to Human Mutations
			26474449	Practical Experience of the Application of a Weighted Burden Test to Whole Exome Sequence Data for Obesity and Schizophrenia
			26244670	The prevalence of melanocortin-4 receptor gene mutations in Slovak obese children and adolescents.
			26229975	How genetic errors in GPCRs affect their function: Possible therapeutic strategies
			26167768	An siRNA-based functional genomics screen for the identification of regulators of ciliogenesis and ciliopathy genes
			25419636	Melanocortin-4 receptor gene variants are not associated with binge-eating behavior in nonobese patients with eating disorders.
			25032700	NCI-60 Whole Exome Sequencing and Pharmacological CellMiner Analyses

			24820477	Genetic Association Study of Adiposity and Melanocortin-4 Receptor (MC4R) Common Variants: Replication and Functional Characterization of Non-Coding Regions
			24705810	The Sum of Many Parts: Potential Mechanisms for Improvement in Glucose Homeostasis After Bariatric Surgery
			24705671	Long-Term Weight-Loss in Gastric Bypass Patients Carrying Melanocortin 4 Receptor Variants
			24705254	Genomic analysis of diffuse intrinsic pontine gliomas identifies three molecular subgroups and recurrent activating ACVR1 mutations
			24611737	Novel variants in the MC4R and LEPR genes among severely obese children from the Iberian population.
			24574081	Hyperphagia: Current Concepts and Future Directions Proceedings of the 2nd International Conference on Hyperphagia
			24385306	The role of common and rare MC4R variants and FTO polymorphisms in extreme form of obesity.
			24276017	Brain-Derived Neurotrophic Factor in Human Subjects with Function-Altering Melanocortin-4 Receptor Variants
			23996627	Family of melanocortin receptor (MCR) genes in mammals:mutations, polymorphisms and phenotypic effects
			23819521	Assessment of computational methods for predicting the effects of missense mutations in human cancers
			23505181	The missense variation landscape of FTO, MC4R and TMEM18 in obese children of African ancestry
			23317811	Obesity surgery: happy with less or eternally hungry?
			23270367	Mutation screen in the GWAS derived obesity gene SH2B1 including functional analyses of detected variants
			23251400	A Novel Melanocortin-4 Receptor Mutation MC4R-P272L Associated with Severe Obesity Has Increased Propensity To Be Ubiquitinated in the ER in the Face of Correct Folding
			23185251	Melanocortin-4 Receptor Mutations and Polymorphisms Do Not Affect Weight Loss after Bariatric Surgery
			23159449	Weight-Independent Effects of Roux-en-Y Gastric Bypass on Glucose Homeostasis via Melanocortin-4 Receptors in Mice and Humans

			23049848	Association between Common Polymorphism near the MC4R Gene and Obesity Risk: A Systematic Review and Meta-Analysis
			22535570	Is there a genetic cause of appetite loss?:an explorative study in 1,853 cancer patients
			22473907	P-selectin genotype is associated with the development of cancer cachexia
			22447289	Loss-of-function mutations in MC4R are very rare in the Greek severely obese adult population.
			22106157	Greater Impact of Melanocortin-4 Receptor Deficiency on Rates of Growth and Risk of Type 2 Diabetes During Childhood Compared With Adulthood in Pima Indians
			22043165	Genetics of Obesity: What have we Learned?
			22043164	Molecular Basis of Obesity: Current Status and Future Prospects
			21976721	The MC4R(I251L) allele is associated with better metabolic status and more weight loss after gastric bypass surgery.
			21934689	Is there a genetic cause for cancer cachexia? - a clinical validation study in 1797 patients
			21404042	Missense mutations and polymorphisms of the MC4R gene in Polish obese children and adolescents in relation to the relative body mass index
			21295023	The genetic epidemiology of melanocortin 4 receptor variants.
			21085626	Investigation of a Genome Wide Association Signal for Obesity: Synthetic Association and Haplotype Analyses at the Melanocortin 4 Receptor Gene Locus
			20587078	Genomic insights into early-onset obesity
			20406574	Melanocortin-4 receptor gene variants in Chilean families: association with childhood obesity and eating behavior.
			20127379	From monogenic to polygenic obesity: recent advances
			19046411	Obesity genes: so close and yet so far...
			18835933	Lower Metabolic Rate in Individuals Heterozygous for Either a Frameshift or a Functional Missense MC4R Variant
			18559663	Prevalence of Melanocortin-4 Receptor Deficiency in Europeans and Their Age-Dependent Penetrance in Multigenerational Pedigrees
			18454148	Common variants near MC4R are associated with fat mass, weight and risk of obesity

			17519222	Non-synonymous polymorphisms in melanocortin-4 receptor protect against obesity: the two facets of a Janus obesity gene.
			17286227	A novel non-synonymous mutation in the melanocortin-4 receptor gene (MC4R) in a 2-year-old Austrian girl with extreme obesity.
			16553946	Prediction of indirect interactions in proteins
			16469222	Melanocortin-4 receptor gene and complications after gastric banding.
			16231025	Screening for melanocortin-4 receptor mutations in a cohort of Belgian morbidly obese adults and children.
			15037865	Binge-eating episodes are not characteristic of carriers of melanocortin-4 receptor gene mutations.
			14764812	Genetic screening for melanocortin-4 receptor mutations in a cohort of Italian obese patients: description and functional characterization of a novel mutation.
			12629567	A novel nonsense mutation in the melanocortin-4 receptor associated with obesity in a Spanish population.
			10199800	Several mutations in the melanocortin-4 receptor gene including a nonsense and a frameshift mutation associated with dominantly inherited obesity in humans.

Supplementary Table 5: All publications regarding *MC4R* variants presented on www.mc4r.org.uk.

Gene	Variant	Amino acid exchange	Number of entries found	Study	Amino Acid Exchange reported	PMID
MC4R	rs121913557	p.V50L	6	unpublished study	V50G	*NA*
				Dubern B, 2001	V50M	11487744
				Lubrano-Berthelier C, 2003	V50M	12499395
				Tao YX, 2003	V50M	12959994
				Xiang Z, 2010	V50M	20462274
				Roubert P, 2010	V50M	20696697
	rs2229616	p.V103I	14	Gu W, 1999	V103I	10078568
				Hinney A, 1999	V103I	10199800
				Ho G, 1999	V103I	10585465
				Jacobson P, 2002	V103I	12364415
				Hinney A, 2003	V103I	12970296
				Xiang Z, 2006	V103I	16752916
				Stutzmann F, 2008	V103I	18559663
				Calton MA, 2009	V103I	19091795
				Thearle MS, 2012	V103I	22106157
				Hatoum IJ, 2012	V103I	22492873
				Melchior C, 2012	V103I	23146882
				Rovite V, 2014	V103I	24385306
				Moore BS, 2014	V103I	24705671
				Brouwers B, 2021	V103I	33761344
	rs13447329	p.T112M	22	Yeo GS, 2003	T112NfsX11	12588803
				Farooqi S, 2003	T112NfsX11	12646665
				Hatoum IJ, 2012	T112NfsX11	22492873
				Hinney A, 2006	T112K	16492696
				Gu W, 1999	T112M	10078568

				Hinney A, 1999	T112M	10199800
				Nijenhuis WA, 2003	T112M	12690102
				Hinney A, 2003	T112M	12970296
				Valli-Jaakola K, 2004	T112M	14764818
				Tao YX, 2005	T112M	16030156
				Hinney A, 2006	T112M	16492696
				Xiang Z, 2006	T112M	16752916
				Stutzmann F, 2008	T112M	18559663
				Calton MA, 2009	T112M	19091795
				Melchior C, 2012	T112M	23146882
				Moore BS, 2014	T112M	24705671
				Brouwers B, 2021	T112M	33761344
				1000Genomes release 17	T112T	*NA*
	rs13447331	p.S127L	15	Lubrano-Berthelier C, 2003	S127L	12499395
				Hinney A, 2003	S127L	12970296
				Valli-Jaakola K, 2004	S127L	14764818
				Govaerts C, 2005	S127L	16083993
				Hinney A, 2006	S127L	16492696
				Xiang Z, 2006	S127L	16752916
				Ahituv N, 2007	S127L	17357083
				Srinivasan S, 2007	S127L	17668051
				Stutzmann F, 2008	S127L	18559663
				Calton MA, 2009	S127L	19091795
				Fan ZC, 2009	S127L	19298524
				Roubert P, 2010	S127L	20696697
				Rouskas K, 2012	S127L	22447289
				Hatoum IJ, 2012	S127L	22492873

				Rovite V, 2014	S127L	24385306
	novel	p.Y153Y	1	ExAC Browser	Y153C	*NA*
	no rsID	p.V193V	1	Hinney A, 1999	V193V	10199800
	rs13447338	p.L211fsX	10	Yeo GS, 1998	L211MfsX6	9771698
				Hinney A, 1999	L211MfsX6	10199800
				Ho G, 1999	L211MfsX6	10585465
				Yeo GS, 2003	L211MfsX6	12588803
				Farooqi S, 2003	L211MfsX6	12646665
				Hinney A, 2003	L211MfsX6	12970296
				Xiang Z, 2006	L211MfsX6	16752916
				Ahituv N, 2007	L211MfsX6	17357083
				Calton MA, 2009	L211MfsX6	19091795
				Lubrano-Berthelier C, 2003	L211P	12851297
	rs582820871	p.I251L	17	Hinney A, 2003	I251WfsX34	12970296
				Lubrano-Berthelier C, 2004	I251WfsX34	15126516
				Tan K, 2009	I251WfsX34	18801902
				Xiang Z, 2010	I251WfsX34	20462274
				Aslan IR, 2011	I251WfsX34	20733581
				van der Berg L, 2011	I251WfsX34	20966905
				Bonnefond A, 2016	I251F	27222505
				Vaisse C, 2000	I251L	10903341
				Hinney A, 2003	I251L	12970296
				Xiang Z, 2006	I251L	16752916
				Thearle MS, 2012	I251L	22106157
				Hatoum IJ, 2012	I251L	22492873
				Melchior C, 2012	I251L	23146882
				Rovite V, 2014	I251L	24385306

				Moore BS, 2014	I251L	24705671
				Brouwers B, 2021	I251L	33761344
				unpublished study	I251T	*NA*

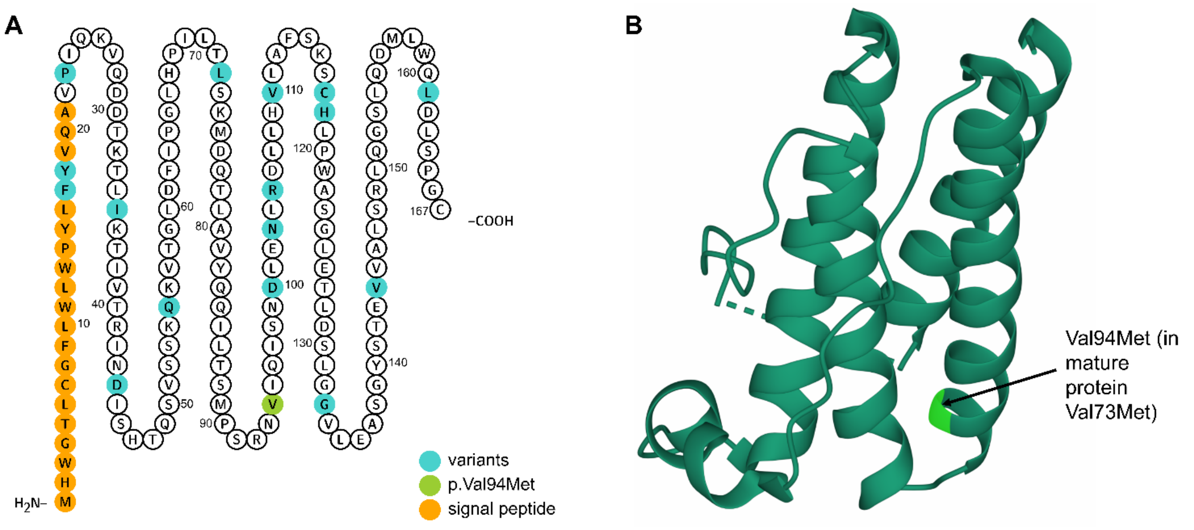

Supplementary Figure 1: Amino acid sequence and 3D structure of leptin. The amino acid sequence of the precursor leptin protein (A) and the 3D structure of the mature leptin protein (B) are represented. In the mature protein, the here detected variant rs17151919 (p.Val94Met) is located on position 73. The amino acid sequence was plotted with the tool Protter[27]. The 3D protein structure was downloaded from the RSC PDB derived from the protein reference 1AX8[28].

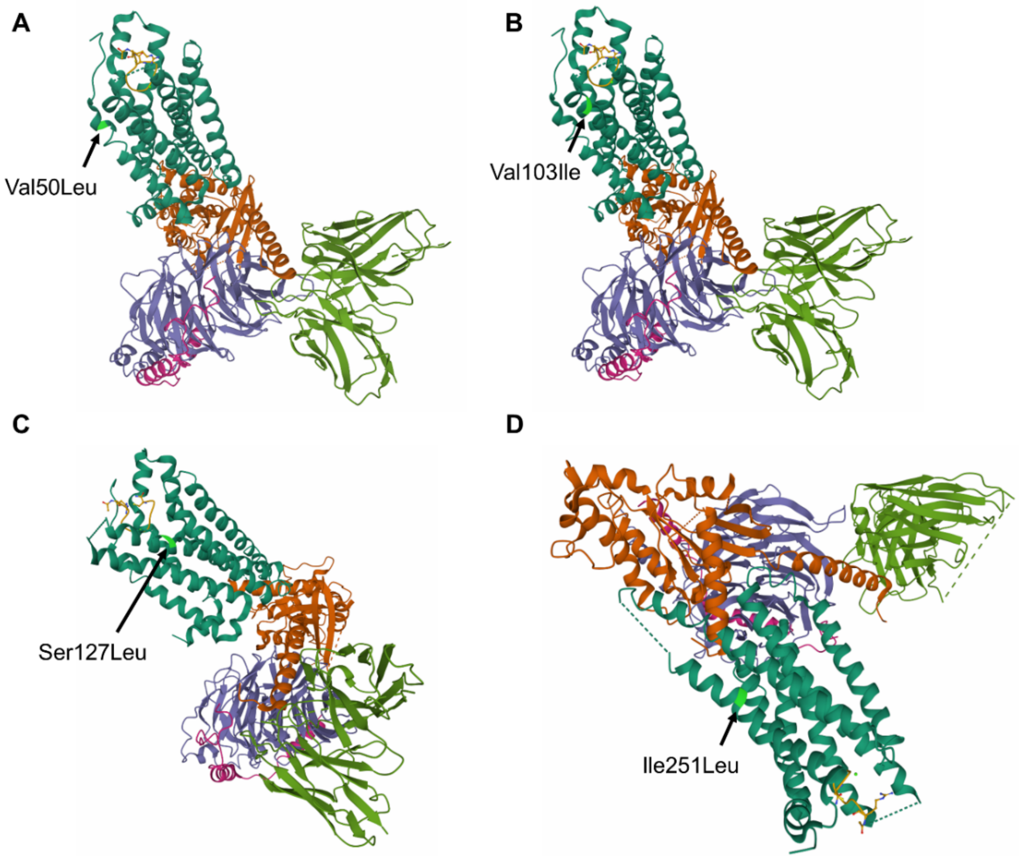

Supplementary Figure 2: 3D structure of the MC4R protein. The here detected *MC4R*-located non-synonymous variants p.Val50Leu (A, rs121913557), p.Val103Ile (B, rs2229616), p.Ser127Leu (C, rs13447331) and p.Ile251Leu (D, rs52820871) are marked in green and indicated by the arrow. The variant p.Thr112Met (rs13447329) could not be visualized as the used reference protein (RSC PDB: 7AUE; [29]) did not contain the corresponding region. To facilitate the visualization, the 3D structures have been rotated around their axis. The 3D structure was downloaded from RSC PDB.

References

1 Allot, A. *et al.* LitVar: a semantic search engine for linking genomic variant data in PubMed and PMC. *Nucleic Acids Res* **46**, W530-W536 (2018). https://doi.org:10.1093/nar/gky355

2 Brouwers, B. *et al.* Human MC4R variants affect endocytosis, trafficking and dimerization revealing multiple cellular mechanisms involved in weight regulation. *Cell Rep* **34**, 108862 (2021). https://doi.org:10.1016/j.celrep.2021.108862

3 Paisdzior, S. *et al.* Differential Signaling Profiles of MC4R Mutations with Three Different Ligands. *Int J Mol Sci* **21** (2020). https://doi.org:10.3390/ijms21041224

4 Lotta, L. A. *et al.* Human Gain-of-Function MC4R Variants Show Signaling Bias and Protect against Obesity. *Cell* **177**, 597-607 e599 (2019). https://doi.org:10.1016/j.cell.2019.03.044

5 Rojo, D., McCarthy, C., Raingo, J. & Rubinstein, M. Mouse models for V103I and I251L gain of function variants of the human MC4R display decreased adiposity but are not protected against a hypercaloric diet. *Mol Metab* **42**, 101077 (2020). https://doi.org:10.1016/j.molmet.2020.101077

6 Geller, F. *et al.* Melanocortin-4 receptor gene variant I103 is negatively associated with obesity. *Am J Hum Genet* **74**, 572-581 (2004). https://doi.org:10.1086/382490

7 Rovite, V. *et al.* The role of common and rare MC4R variants and FTO polymorphisms in extreme form of obesity. *Mol Biol Rep* **41**, 1491-1500 (2014). https://doi.org:10.1007/s11033-013-2994-4
8 Rong, R. *et al.* Identification and functional characterization of three novel human melanocortin-4 receptor gene variants in an obese Chinese population. *Clin Endocrinol (Oxf)* **65**, 198-205 (2006). https://doi.org:10.1111/j.1365-2265.2006.02573.x
9 Melchior, C. *et al.* Clinical and functional relevance of melanocortin-4 receptor variants in obese German children. *Horm Res Paediatr* **78**, 237-246 (2012). https://doi.org:10.1159/000343816
10 Gu, W. *et al.* Identification and functional analysis of novel human melanocortin-4 receptor variants. *Diabetes* **48**, 635-639 (1999). https://doi.org:10.2337/diabetes.48.3.635
11 Ho, G. & MacKenzie, R. G. Functional characterization of mutations in melanocortin-4 receptor associated with human obesity. *J Biol Chem* **274**, 35816-35822 (1999). https://doi.org:10.1074/jbc.274.50.35816
12 Hinney, A. *et al.* Melanocortin-4 receptor gene: case-control study and transmission disequilibrium test confirm that functionally relevant mutations are compatible with a major gene effect for extreme obesity. *J Clin Endocrinol Metab* **88**, 4258-4267 (2003). https://doi.org:10.1210/jc.2003-030233
13 Xiang, Z. *et al.* Pharmacological characterization of 40 human melanocortin-4 receptor polymorphisms with the endogenous proopiomelanocortin-derived agonists and the agouti-related protein (AGRP) antagonist. *Biochemistry* **45**, 7277-7288 (2006). https://doi.org:10.1021/bi0600300
14 Thearle, M. S. *et al.* Greater impact of melanocortin-4 receptor deficiency on rates of growth and risk of type 2 diabetes during childhood compared with adulthood in Pima Indians. *Diabetes* **61**, 250-257 (2012). https://doi.org:10.2337/db11-0708
15 He, S. & Tao, Y. X. Defect in MAPK signaling as a cause for monogenic obesity caused by inactivating mutations in the melanocortin-4 receptor gene. *Int J Biol Sci* **10**, 1128-1137 (2014). https://doi.org:10.7150/ijbs.10359
16 Tao, Y. X. & Segaloff, D. L. Functional analyses of melanocortin-4 receptor mutations identified from patients with binge eating disorder and nonobese or obese subjects. *J Clin Endocrinol Metab* **90**, 5632-5638 (2005). https://doi.org:10.1210/jc.2005-0519
17 Valli-Jaakola, K. *et al.* Identification and characterization of melanocortin-4 receptor gene mutations in morbidly obese finnish children and adults. *J Clin Endocrinol Metab* **89**, 940-945 (2004). https://doi.org:10.1210/jc.2003-031182
18 Nijenhuis, W. A., Garner, K. M., van Rozen, R. J. & Adan, R. A. Poor cell surface expression of human melanocortin-4 receptor mutations associated with obesity. *J Biol Chem* **278**, 22939-22945 (2003). https://doi.org:10.1074/jbc.M211326200
19 Fan, Z. C. & Tao, Y. X. Functional characterization and pharmacological rescue of melanocortin-4 receptor mutations identified from obese patients. *J Cell Mol Med* **13**, 3268-3282 (2009). https://doi.org:10.1111/j.1582-4934.2009.00726.x
20 Falls, B. A. & Zhang, Y. Insights into the Allosteric Mechanism of Setmelanotide (RM-493) as a Potent and First-in-Class Melanocortin-4 Receptor (MC4R) Agonist To Treat Rare Genetic Disorders of Obesity through an in Silico Approach. *ACS Chem Neurosci* **10**, 1055-1065 (2019). https://doi.org:10.1021/acschemneuro.8b00346
21 Srinivasan, S., Santiago, P., Lubrano, C., Vaisse, C. & Conklin, B. R. Engineering the melanocortin-4 receptor to control constitutive and ligand-mediated G(S) signaling in vivo. *PLoS One* **2**, e668 (2007). https://doi.org:10.1371/journal.pone.0000668
22 Mo, X. L. & Tao, Y. X. Activation of MAPK by inverse agonists in six naturally occurring constitutively active mutant human melanocortin-4 receptors. *Biochim Biophys Acta* **1832**, 1939-1948 (2013). https://doi.org:10.1016/j.bbadis.2013.06.006
23 Xiang, Z. *et al.* Peptide and small molecules rescue the functional activity and agonist potency of dysfunctional human melanocortin-4 receptor polymorphisms. *Biochemistry* **46**, 8273-8287 (2007). https://doi.org:10.1021/bi7007382

24 Lubrano-Berthelier, C. *et al.* Intracellular retention is a common characteristic of childhood obesity-associated MC4R mutations. *Hum Mol Genet* **12**, 145-153 (2003). https://doi.org:10.1093/hmg/ddg016

25 Granell, S. *et al.* A novel melanocortin-4 receptor mutation MC4R-P272L associated with severe obesity has increased propensity to be ubiquitinated in the ER in the face of correct folding. *PLoS One* **7**, e50894 (2012). https://doi.org:10.1371/journal.pone.0050894

26 Prusis, P., Uhlen, S., Petrovska, R., Lapinsh, M. & Wikberg, J. E. Prediction of indirect interactions in proteins. *BMC Bioinformatics* **7**, 167 (2006). https://doi.org:10.1186/1471-2105-7-167

27 Omasits, U., Ahrens, C. H., Muller, S. & Wollscheid, B. Protter: interactive protein feature visualization and integration with experimental proteomic data. *Bioinformatics* **30**, 884-886 (2014). https://doi.org:10.1093/bioinformatics/btt607

28 Zhang, F. *et al.* Crystal structure of the obese protein leptin-E100. *Nature* **387**, 206-209 (1997). https://doi.org:10.1038/387206a0

29 Israeli, H. *et al.* Structure reveals the activation mechanism of the MC4 receptor to initiate satiation signaling. *Science* **372**, 808-814 (2021). https://doi.org:10.1126/science.abf7958
